# Exome sequencing of a family with lone, autosomal dominant atrial flutter identifies a rare variation in ABCB4 significantly enriched in cases

**DOI:** 10.1186/s12863-015-0177-0

**Published:** 2015-02-11

**Authors:** Anna Maciąg, Francesco Villa, Anna Ferrario, Chiara Carmela Spinelli, Albino Carrizzo, Alberto Malovini, Annalaura Torella, Chiara Montenero, Attilio Parisi, Gianluigi Condorelli, Carmine Vecchione, Vincenzo Nigro, Annibale Sandro Montenero, Annibale Alessandro Puca

**Affiliations:** IRCCS MultiMedica, Milano, Italy; ITB-CNR, Segrate, MI Italy; IRCCS Neuromed, Parco Tecnologico, Pozzilli, IS Italy; Università di Pavia, Pavia, Italy; Seconda Università degli Studi di Napoli, Napoli, Italy; Università degli Studi di Roma Tor Vergata, Roma, Italy; Università degli Studi di Roma “Foro Italico”, Rome, Italy; Humanitas Clinical and Research Center, Rozzano, MI Italy; Università degli Studi di Milano, Milan, Italy; Università degli Studi di Salerno, Salerno, Italy

**Keywords:** Pedigree, Atrial flutter, Atrial fibrillation, SNPs, Exome-sequencing, ATP-binding cassette B4 (ABCB4)

## Abstract

**Background:**

Lone atrial flutter (AFL) and atrial fibrillation (AF) are common and sometimes consequential cardiac conduction disorders with a strong heritability, as underlined by recent genome-wide association studies that identified genetic modifiers. Follow-up family-based genetic analysis also identified Mendelian transmission of disease alleles. Three affected members were exome-sequenced for the identification of potential causative mutations, which were subsequently validated by direct sequencing in the other 3 affected members. Taqman assay was then used to confirm the role of any mutation in an independent population of sporadic lone AFL/AF cases.

**Results:**

The family cluster analysis provided evidence of genetic inheritance of AFL in the family via autosomal dominant transmission. The exome-sequencing of 3 family members identified 7 potential mutations: of these, rs58238559, a rare missense genetic variant in the ATP-binding cassette sub-family B, member 4 (*ABCB4*) gene was carried by all affected members. Further analysis of 82 subjects with sporadic lone AF, 63 subjects with sporadic lone AFL, and 673 controls revealed that the allele frequency for this variation was significantly higher in cases than in the controls (0.05 vs. 0.01; OR = 3.73; 95% CI = 1.16–11.49; *P* = 0.013).

**Conclusions:**

rs58238559 in *ABCB4* is a rare missense variant with a significant effect on the development of AFL/AF.

**Electronic supplementary material:**

The online version of this article (doi:10.1186/s12863-015-0177-0) contains supplementary material, which is available to authorized users.

## Background

Atrial flutter (AFL) is the second most common arrhythmia after atrial fibrillation (AF). It is a heart rhythm disturbance that results in the upper chambers of the heart beating up to five-times faster (usually 240–350 atrial contractions/minute) than normal. There is a close interrelationship between AFL and AF, with AFL of variable duration preceding the onset of AF in many instances [1].

AFL is significantly associated with alcohol intake in patients under 60 years of age [2]. A study based on a US population determined that the incidence rates for AFL ranged from 5 per 100,000 in those less than 50 years old to 587 per 100,000 in those older than 80 years old [3]. Moreover, AFL was 2.5-times more common in men, and the risk of developing AFL increased with concomitant hyperthyroidism, valvular diseases, myocardial infarction, and congestive heart failure [4].

Hypertension and diabetes were the only significant independent cardiovascular risk factors for AF when controlling for age and other predisposing conditions in 38-year follow-up data from the Framingham Study [5]. Other evidence suggested that AF in parents increases the future risk of AF in their offspring and that the risk increased when the parent developed AF before age 75 years [4].

Genome-wide association studies (GWAS) have been recently conducted for AFL/AF. The first GWAS identified a genetic modifier for AF and AFL in an Icelandic population [6]: it found an association for AFL/AF with the rs2200733 polymorphism in the *PITX2* gene on chromosome 4q25 [odds ratio (OR), 1.75; *P* = 1.6×10^-9^]. This association was replicated in other populations, including the population we are investigating [7]. Others studies identified variants in *ZFHX3* [8], *KCNN3* [9], *SCN5A* [10], and a further six loci [11] associated with AF. All these studies interrogated hundreds of thousands to millions of common polymorphisms genome-wide with the potential of impacting the AF phenotype, but with only a small effects size [12]. These variants explain only a small percentage of the high heritability estimated for AF. The presence of rare variants with large effects size are not sufficiently covered by GWAS, and this could explain the missing heritability of AF [12].

Thus, genetic analysis on familial clusters of AFL/AF could disclose rare variants with large size effects [13]. Several studies have reported rare variants in familial cases of AF in genes encoding cardiac gap junctions, signaling molecules, and ion channels, supporting a role for cardiac depolarization–repolarization in susceptibility to AF. Most AF-related genes encode potassium and sodium channels [13]. Of these, mutations in the sodium ion channel gene *SCN5A*, at 3p22.2 (OMIM#*600163), have been correlated with idiopathic AF [14] and possibly also with AFL, conduction diseases, Brugada syndrome, and sudden cardiac death [15]. Among the potassium channel genes, *KCNQ1* (OMIM#*607542), previously identified as causative for long and short QT syndromes, has been also identified as responsible for a familial form of AF [16].

Here, we report a study on a pedigree containing 6 AFL-affected family members. Exome sequencing of 3 affected individuals, followed by direct sequencing of the other affected members and of 3 unaffected members, indicated a possible causative role of a missense mutation in the ATP-binding cassette sub-family B, member 4 gene (*ABCB4*). This mutation was significantly enriched in sporadic lone AFL/AF cases when compared with a control population.

## Results

### Pedigree

**Figure 1:**
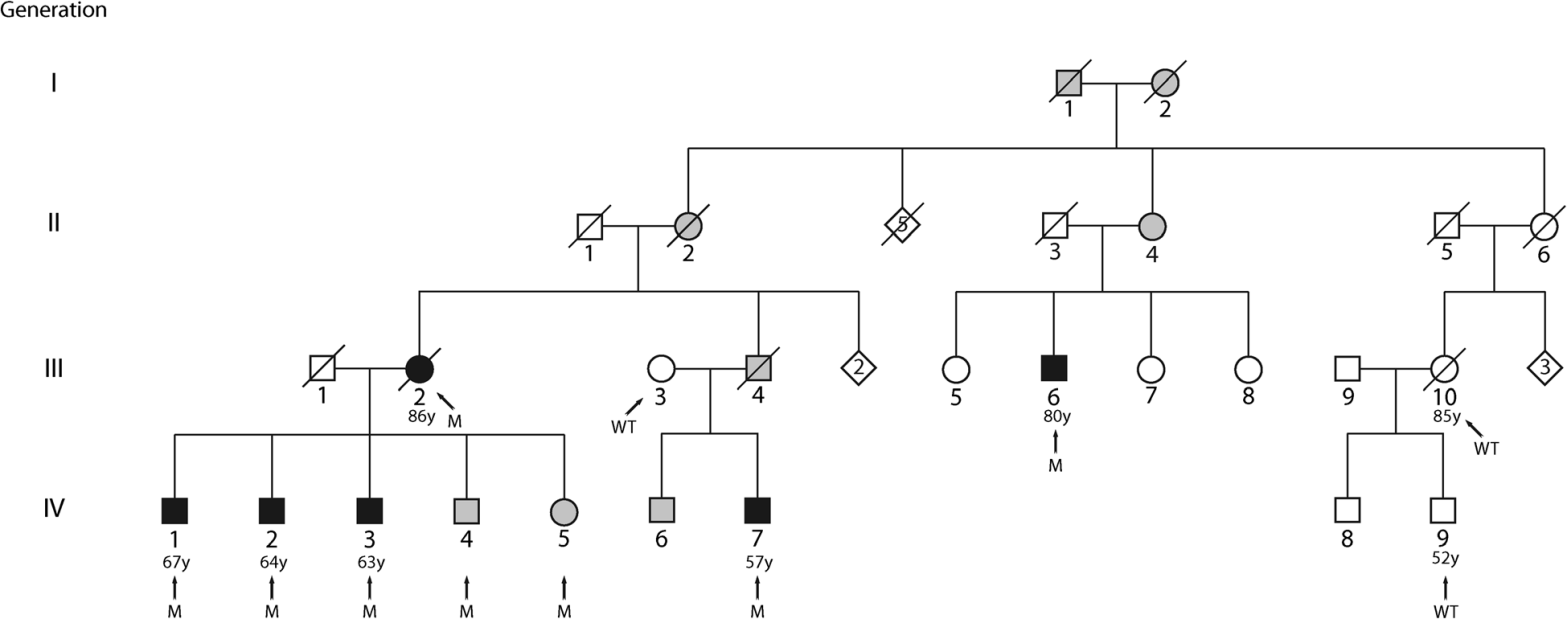
**Pedigree of the family affected by atrial flutter** **/** **fibrillation** **.** Generation is indicated with roman numerals, and individual ID is indicated with Arabic numerals. Solid squares (males) and circles (females) indicate affected subjects, open symbols indicate unaffected subjects, and gray symbols indicate unknown disease status. Diamonds indicate not-relevant subjects and the numbers within them are the number of subjects. Diagonal lines indicate dead subjects. Arrows identify the analyzed subjects. WT indicates homozygotic carriers of rs58238559 major allele while M indicates the heterozygotic subjects for rs58238559 variant. Ages are given beneath the individual’s ID number.

The presentation of 3 brothers (subject IDs: IV:1, IV:2, and IV:3) for treatment of AFL led to the construction of a four-generation pedigree (Figure 1) [17]. The pedigree contained 43 subjects (23 males and 20 females). A brief medical history was obtained for each subject (where possible) in order to identify known risk factors for AFL [3]. A summary of the subjects with documented cardiovascular abnormalities is provided in Table 1 (cases are described in the Methods section).

**Table 1:** **Summary of Pedigree Members with Documented Cardiovascular Abnormalities**

**Generation**	**Subject ID**	**Age**	**Sex**	**Risk factor**	**Cardiovascular abnormality**
III	2	86	Female	Hypertension	Atrial flutter
III	6	80	Male	Hypertension; alcohol abuse	Atrial flutter
IV	1	67	Male	Hypertension; smoking	Atrial flutter
IV	2	64	Male	Smoking	Atrial flutter
IV	3	63	Male	Smoking	Atrial flutter
IV	7	57	Male	---	Atrial flutter
IV	9	52	Male	---	Myocardial infarction

No direct parent-to-child transmission of AFL has been documented in the literature. However, in the present case study, AFL was identified in a woman (III:2) and in 3 (60%) of her 5 children (IV:1, IV:2, and IV:3). AFL was also documented in a nephew (IV:7) and a cousin (III:6). The father of IV:7 died from a cerebral ictus at 49 years of age, possibly as a complication of AFL. Furthermore, AFL was documented in the 3^rd^ and 4^th^ generations only, likely because of poor diagnosis and inadequate reporting of this dysrhythmia in earlier generations. The almost complete absence of any known risk factor(s) for AFL [3] other than hypertension in 3 (50%; III:2, III:6, and IV:1) of the 6 subjects with AFL suggested that this family has a heritable susceptibility for AFL. The idiopathy plus familial clustering of this dysrhythmia are compatible with autosomal dominant genetic transmission.

### Exome sequencing in familial AFL cases

To identify the causative gene mutation, we sequenced the whole exome of 3 affected subjects of the family, belonging to the third (III:6) and fourth (IV:1 and IV:7) generations (complete data sets of exome sequencing results are available in the on-line Additional file 1). The filtered results indicated that: a) none of the previously identified causative genes harbored rare missense mutations in the 3 sequenced subjects; and b) 6 damaging, non-synonymous single-nucleotide variations (SNVs) and 1 stop–gain mutation were shared by the 3 subjects (Table 2).

**Table 2:** **SNVs Identified by Exome Sequencing and Validated by PCR Products Sequencing**

**Variant coordinates**	**SNP ID**	**Gene**	**Variation**	**Nucleotide changes**	**MAF** * (%)
chr7:123599641	rs199703625	*SPAM1*	non-synonymous SNV	A-- > G	0.100
chr11:1769211	---	*IFITM10*	non-synonymous SNV	C-- > T	---
chr7:81601108	rs78086631	*CACNA2D1*	non-synonymous SNV	G-- > A	0.270
chr7:87082273	rs58238559	*ABCB4*	non-synonymous SNV	A-- > G	0.652
chr15:76225153	---	*FBXO22*	non-synonymous SNV	C-- > A	---
chr11:117257921	---	*CEP164*	non-synonymous SNV	G-- > T	---
chr2:27522165	rs146448995	*TRIM54*	stop–gain mutation	G-- > T	0.022

*UCSC Genome Browser database. MAF = minor allele frequency.

**Figure 2:**
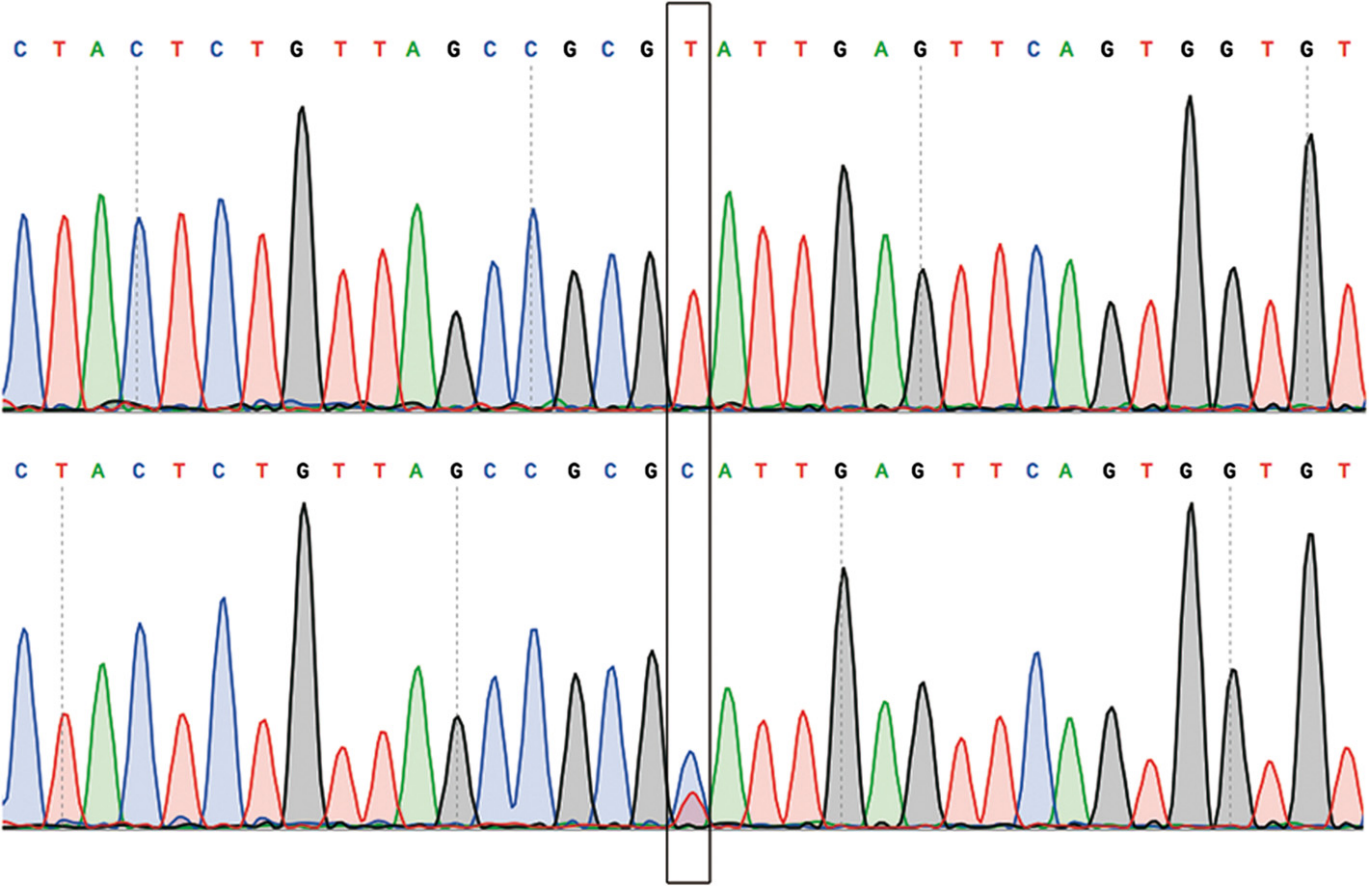
**Sequencing electropherograms** **.** Electropherograms of control (upper) and atrial flutter/fibrillation-affected (lower) subjects with rs58238559 in the *ABCB4* gene. Heterozygosity is indicated by the presence of two peaks corresponding to T and C (in the box).

We checked the involvement of these mutations in AFL by sequencing the DNA of the other affected members (III:2, IV:2, and IV:3) and of 3 unaffected members (III:3; III:10 and IV:9) of the family. This additional sequencing analysis was conducted because of the small number of subjects analyzed by exome sequencing. Of the 7 variations identified, only rs58238559 (A599G; NM_000443.3) in the *ABCB4* gene was in heterozygosity in all affected individuals (Figure 2). The father (III:4) of affected individual IV:7 – who died of a cerebral ictus at 49 years of age, possibly as a complication of AFL – carried a copy of the minor allele (G) of rs58238559 (his living, healthy wife is wild type, and the affected child is a G carrier), so he probably transmitted AFL to one of his two children. Of note, descendants of II:6 (III:10 and IV:9), whose family branch is not affected by the disease, are not rs58238559 carriers. On the other hand, IV:4 and IV:5, who are offspring of III:2 and who have no manifestation of the disease (as yet), carry the rs58238559 minor allele in heterozygosity. This could explain the variable onset of disease.

The rs58238559 single-nucleotide polymorphism (SNP) is located in the *ABCB4* gene on chr7:87082273, and determines the nucleotide variation A599G (NM_000443.3) (Figure 2), producing the amino acid change Thr175Ala (NP_000434.1). Of note, a Thr175Val variation at the same position has been previously related to gallbladder disease in a sporadic case [18], while *ABCB4* mutations are usually associated with familial forms of the disease [19]. The medical history of the AFL-affected pedigree did not disclose any gallbladder disease. Taken together, the above data leads to the speculation that ABCB4 variants at position 175 produce a modest genetic predisposition for gallbladder disease, whereas Thr175Ala produces a familial autosomal form of lone AFL.

### AFL/AF case–control validation

To corroborate our finding on the role of the *ABCB4* gene variation in AFL/AF, we analyzed a cohort of AFL/AF cases and controls, part of which we previously used to validate rs2200733 in AFL/AF [7]. The criteria of adopting AFL/AF cases comes from previous evidences of shared genetic risk factors, despite are two distinct clinical entities. No deviations from the Hardy–Weinberg equilibrium were observed for the analyzed marker (p HWD = 1). We found that the frequency of the mutated allele was significantly higher in cases than in the controls (0.05 vs. 0.01; OR = 3.73; 95% confidence interval =1.16–11.49; *P* = 0.013). Of note, there was sufficient statistical power to detect the reported association, according to the frequencies in cases and controls and sample sizes observed (1-β = 0.81). With respect to the rest of the cohort, individuals with the Thr175Ala amino acid change in ABCB4 have a 3.75-fold increase in the probability of developing atrial fibrillation/flutter. We also repeated the analysis separating AFL from AF to evaluate their contribution to the association and we found very similar carrier frequencies in the two cohorts (AFL N = 63; 4,76% carriers, AF N = 78; 4,88% carriers), indicating a similar genetic effect in the two populations.

### Limitations

Despite the filtering cut off that we adopted of MAF > 1% is relatively high and would increase the chance of false positive results [20] we believe to have avoided this limitation by replicating the data in an independent cohort. One additional limitation is the lack of covariates in our analysis, as for drinking behavior, even if we are not aware of any bias in recruiting cases and controls (i.e. both arms of the study should share same habits).

## Discussion

Arrhythmia of the atria is a common disorder with an important impact on morbidity and mortality [21]. It increases tremendously the risk of complications, such as pulmonary embolism and stroke. Understanding the underlying molecular mechanisms of AFL/AF is, therefore, very important. To this end, genetic approaches aimed at uncovering the pathogenesis of this disease are very useful. A recent GWAS identified common polymorphisms able to modify the risk of AF, while family-based studies have disclosed many mutations causative of familial and sporadic forms of lone AF [13].

The present study of a family with a strong clustering of AFL-affected members has found that the rs58238559 SNP in *ABCB4*, which produces a Thr175Ala amino acid change, is associated with AFL/AF. Moreover, follow-up analysis has found significant enrichment of rs58238559 in sporadic AFL/AF cases. Thus, we propose *ABCB4* as a previously unrecognized disease-related gene for lone AF/AFL, and that it should be further investigated in relation to AFL/AF epidemiology and pathophysiology.

Functionally, ABCB4 belongs to a family of lipid transporters and is specifically involved in transport of phosphatidylcholine (PC) across membranes [22]. ABCB4 is mainly expressed in liver, but also in heart, adrenal gland, striated muscle, spleen, and tonsil [23]. Interestingly, ABCB4^-/-^ knock-out mice had low secretion of PC into the bile, leading to cholestasis and liver fibrosis [24]. In humans, ABCB4 associates with progressive familial intrahepatic cholestasis [19], and intrahepatic cholestasis during pregnancy is a common disorder associated with fetal AF [25]. Thus, we propose a pathogenic mechanism whereby the mutation of *ABCB4* generates AF/AFL either directly in the heart or indirectly through the liver via altered PC transport. A common genetic origin of cholestasis and AF could also explain why cholestasis has been reported in AF patients under therapy [26-28].

## Conclusions

We have found genetic evidence for a role of ABCB4 in familial lone AF and sporadic lone AFL/AF. This is supported by pre-existing data on the role of ABCB4 in cholestasis and by the correlation of cholestasis with fetal arrhythmia and adult AF/AFL. Further genetic analyses in humans as well as cardiac phenotype characterization of the ABCB4^-/-^ mouse will better clarify ABCB4’s role in AF/AFL.

## Methods

### Subject characteristics

The unusual presentation of 3 brothers (subject IDs: IV:1, IV:2, and IV:3 described below) for treatment of AFL led to the construction of a four-generation pedigree (Figure 1) [17]. The pedigree contained 43 subjects (23 men and 20 women). A brief medical history was obtained for each subject (where possible) in order to identify known risk factors for AFL [3].

Subject IV:1 A 67-year-old male diagnosed with common AFL approximately 5 years ago. Medical history identified the presence of hypertension and a history of smoking. Absence of structural anomalies in the heart or cardiac disease suggested lone AFL. This subject was ineffectively managed by antiarrhythmic (AA) drug therapy (flecainide and enalapril), but recently underwent catheter ablation to create bi-directional conduction block across the cavotricuspid isthmus (CTI). The subject continues to be arrhythmia free following the catheter ablation procedure.

Subject IV:2 A 64-year-old male diagnosed with common AFL approximately 1 year ago. Apart from smoking, medical history did not identify the presence of any chronic conditions or risk factors for AFL. Absence of structural anomalies in the heart or cardiac disease suggested lone AFL. This subject was ineffectively managed by AA drug therapy (flecainide and sotalol), but recently underwent catheter ablation to create bi-directional conduction block across the CTI. The subject continues to be arrhythmia free following the catheter ablation procedure.

Subject IV:3 A 63-year-old male diagnosed with common AFL approximately 1 year ago. Apart from smoking, medical history did not identify the presence of any chronic conditions or risk factors for AFL. Absence of structural anomalies in the heart or cardiac disease suggested lone AFL. This subject was ineffectively managed by AA drug therapy (propafenone), but recently underwent catheter ablation to create bi-directional conduction block across the CTI. The subject continues to be arrhythmia free following the catheter ablation procedure.

Subject IV:7 A 57-year-old male diagnosed with common AFL approximately 4 years ago. Medical history did not identify the presence of any chronic conditions or risk factors for AFL. This subject continues to be reasonably managed by AA drug therapy alone (flecainide), but continues to experience arrhythmic symptoms.

Subject III:2 The 86-year-old mother of IV:1, IV:2, and IV:3 also has AFL, which was diagnosed approximately 25 years ago. Medical history identified hypertension, but no other risk factors. Of the 5 offspring of this subject, 3 (60%) developed AFL by the 6^th^ decade of life.

Subject III:6 An AFL-affected cousin of III:2. Medical history for this cousin identified excessive alcohol consumption and hypertension as risk factors.

In addition, 145 sporadic AFL/AF patients (N = 63 with AFL, and N = 82 with AF) were enrolled for a case–control validation study. To this end, consecutive symptomatic AF and common AFL patients referred to our institution for electrophysiological studies and catheter ablation were enrolled. The patients were anti-arrhythmic-drugs-free a week before admission. The study was conducted in accordance with the ethical principles that have their origins in the Declaration of Helsinki. Written informed consent was obtained from all participants for the current study that was approved by the IRCCS MultiMedica Review Board CE/CE/92/2013/LDC. Written consent form for data publication was obtained from all involved subjects. Participants were evaluated by medical history, physical examination, and electrocardiogram (ECG). Clinical assessment was performed without knowledge of genotype.

### Exome sequencing

Exomic regions of genomic DNA of 3 affected pedigree subjects (subject IDs: III:6, IV:1, and IV:7) were enriched using either the TruSeq™ Exome Enrichment Kit (Illumina) or the Agilent Haloplex Exome kit based on DNA digestion and capture. Exomes were barcoded and sequenced at multiple sites on the Illumina HiSeq1000 platform, and either 2 × 76-bp (TruSeq) or 2 × 100-bp (Haloplex) PE libraries, using TruSeq SBS Kit v3–HS (Illumina) reagents and a TruSeq PE Cluster kit v.3-cbot-HS flow cell. Average coverage for all the experiments was 70x and at least 20× for 89% of the target. Paired sequencing reads were aligned to the reference genome (UCSC, hg19 build) using BWA [29] and sorted with SAMtools [30] and Picard (http://broadinstitute.github.io/picard/). Post-alignment processing (local realignment around insertions-deletions and base recalibration), SNV, and small insertions-deletions (ins-del) calling were performed with Genome Analysis Toolkit (GATK) [31] with parameters adapted to the haloplex-generated sequences. The called SNV and ins-del variants produced with both platforms were annotated using ANNOVAR [32].

### Data filtering

The results were first filtered to eliminate common variants (MAF > 1%), variants with low quality score, and variants not shared by all analyzed affected subjects, when covered. Additional frequency filters were used by comparing internal databases of whole exome sequencing data (n = 300). Prioritization was also made based on MAF frequency.



### Validation analysis

Genomic DNA of the remaining affected subjects and of 3 unaffected subjects was amplified with polymerase chain reaction (PCR), following standard methods, for selected single-nucleotide polymorphisms (SNPs). The amplified fragments were purified with Wizard SV Gel and PCR Clean-Up System (Promega) and were sequenced to identify mutations associated with AFL. Primer sequences and amplification temperatures are listed in Table 3.

**Table 3:** **Primer Sequences Used in the Validation Analysis**

**Gene**	**Forward Primer** **(** **5** **′-** **3** **′)**	**Reverse Primer** **(** **5** **′-** **3** **′)**	**Tm** **(°** **C** **)**
*SPAM1*	CAGAAATCTTGCTTGCTCCTAG	TTCAAGTGTCGGTTTTCCAC	58
*IFITM10*	CAGCACCACGGACGGC	GGCAGGGGGCTTGGAC	64
*CACNA2D1*	CTGTGTTAGGTAACGCGGAT	CTGAAAAACACCCACAACTG	57
*ABCB4*	CTGCTAGACATGGCTGCCAG	TTCATTTTGGACTTTGGCAGC	62
*FBXO22*	CCTCTGGATATTGATGCCTC	CTTTCTAAATGCATCAGCCTC	57
*CEP164*	TCTTTGACTCCTGATTGTGGG	CTCTTGCTTGGATTCCAGCAG	61
*TRIM54*	TTCATGCTTAAGGTCCACCTC	ACAGTCCTTGTGGGCACCGAAG	63

### Taqman assay

DNA was extracted from peripheral blood (QIAamp DNA Blood Midi kit, Qiagen) and genotyped with Taqman assays on an ABI 7900HT Real Time PCR (Applied Biosystems). For the screening, we used a probe for rs58238559. The reactions were performed using Genotyping Master Mix (Applied Biosystems). Data analysis was performed with Sequence Detection Systems (Applied Biosystems).

### Statistics for case–control analysis

Differences in genotype distribution between cases and controls were tested with two-sided Fisher’s Exact test, as implemented in the R statistical software tool (http://www.r-project.org/). Deviations from the Hardy–Weinberg equilibrium were tested with the PLINK software tool [33]. The threshold for identifying statistically significant associations was set at a *P*-value < 0.05.

## Additional file

Additional file 1:
**Complete data sets: the file lists the results of exome sequencing analysis.**

## Abbreviations

AA: Antiarrhythmic
AF: atrial fibrillation
AFL: Atrial flutter
CI: Confidence interval
CTI: Cavotricuspid isthmus
ECG: Electrocardiogram
GWAS: Genome-wide association studies
MAF: Minor allele frequency
PC: Phosphatidylcholine

## References

[1] Waldo AL, Feld GK (2008). Inter-relationships of atrial fibrillation and atrial flutter mechanisms and clinical implications. J Am Coll Cardiol.

[2] Marcus GM, Smith LM, Whiteman D, Tseng ZH, Badhwar N, Lee BK (2008). Alcohol intake is significantly associated with atrial flutter in patients under 60 years of age and a shorter right atrial effective refractory period. Pacing Clin Electrophysiol.

[3] Granada J, Uribe W, Chyou PH, Maassen K, Vierkant R, Smith PN (2000). Incidence and predictors of atrial flutter in the general population. J Am Coll Cardiol.

[4] Fox CS, Parise H, D’Agostino RB, Lloyd-Jones DM, Vasan RS, Wang TJ (2004). Parental atrial fibrillation as a risk factor for atrial fibrillation in offspring. JAMA.

[5] Aksnes TA, Schmieder RE, Kjeldsen SE, Ghani S, Hua TA, Julius S (2008). Impact of new-onset diabetes mellitus on development of atrial fibrillation and heart failure in high-risk hypertension (from the VALUE Trial). Am J Cardiol.

[6] Gudbjartsson DF, Arnar DO, Helgadottir A, Gretarsdottir S, Holm H, Sigurdsson A (2007). Variants conferring risk of atrial fibrillation on chromosome 4q25. Nature.

[7] Viviani Anselmi C, Novelli V, Roncarati R, Malovini A, Bellazzi R, Bronzini R (2008). Association of rs2200733 at 4q25 with atrial flutter/fibrillation diseases in an Italian population. Heart.

[8] Benjamin EJ, Rice KM, Arking DE, Pfeufer A, van Noord C, Smith AV (2009). Variants in ZFHX3 are associated with atrial fibrillation in individuals of European ancestry. Nat Genet.

[9] Ellinor PT, Lunetta KL, Glazer NL, Pfeufer A, Alonso A, Chung MK (2010). Common variants in KCNN3 are associated with lone atrial fibrillation. Nat Genet.

[10] Olesen MS, Yuan L, Liang B, Holst AG, Nielsen N, Nielsen JB (2012). High prevalence of long QT syndrome-associated SCN5A variants in patients with early-onset lone atrial fibrillation. Circ Cardiovasc Genet.

[11] Ellinor PT, Lunetta KL, Albert CM, Glazer NL, Ritchie MD, Smith AV (2012). Meta-analysis identifies six new susceptibility loci for atrial fibrillation. Nat Genet.

[12] Sinner MF, Ellinor PT, Meitinger T, Benjamin EJ, Kaab S (2011). Genome-wide association studies of atrial fibrillation: past, present, and future. Cardiovasc Res.

[13] Olesen MS, Nielsen MW, Haunso S, Svendsen JH (2014). Atrial fibrillation: the role of common and rare genetic variants. Eur J Hum Genet.

[14] Olson TM, Alekseev AE, Liu XK, Park S, Zingman LV, Bienengraeber M (2006). Kv1.5 channelopathy due to KCNA5 loss-of-function mutation causes human atrial fibrillation. Hum Mol Genet.

[15] Rossenbacker T, Carroll SJ, Liu H, Kuiperi C, de Ravel TJ, Devriendt K (2004). Novel pore mutation in SCN5A manifests as a spectrum of phenotypes ranging from atrial flutter, conduction disease, and Brugada syndrome to sudden cardiac death. Heart Rhythm.

[16] Chen YH, Xu SJ, Bendahhou S, Wang XL, Wang Y, Xu WY (2003). KCNQ1 gain-of-function mutation in familial atrial fibrillation. Science.

[17] Bennett RL, Steinhaus KA, Uhrich SB, O'Sullivan CK, Resta RG, Lochner-Doyle D (1995). Recommendations for standardized human pedigree nomenclature. Pedigree Standardization Task Force of the National Society of Genetic Counselors. Am J Hum Genet.

[18] Rosmorduc O, Hermelin B, Poupon R (2001). MDR3 gene defect in adults with symptomatic intrahepatic and gallbladder cholesterol cholelithiasis. Gastroenterology.

[19] de Vree JM, Jacquemin E, Sturm E, Cresteil D, Bosma PJ, Aten J (1998). Mutations in the MDR3 gene cause progressive familial intrahepatic cholestasis. Proc Natl Acad Sci U S A.

[20] Norton N, Robertson PD, Rieder MJ, Zuchner S, Rampersaud E, Martin E (2012). Evaluating pathogenicity of rare variants from dilated cardiomyopathy in the exome era. Circ Cardiovasc Genet.

[21] Benjamin EJ, Wolf PA, D’Agostino RB, Silbershatz H, Kannel WB, Levy D (1998). Impact of atrial fibrillation on the risk of death: the Framingham Heart Study. Circulation.

[22] van Helvoort A, Smith AJ, Sprong H, Fritzsche I, Schinkel AH, Borst P (1996). MDR1 P-glycoprotein is a lipid translocase of broad specificity, while MDR3 P-glycoprotein specifically translocates phosphatidylcholine. Cell.

[23] Smit JJ, Schinkel AH, Mol CA, Majoor D, Mooi WJ, Jongsma AP (1994). Tissue distribution of the human MDR3 P-glycoprotein. Lab Invest.

[24] Smit JJ, Schinkel AH, Oude Elferink RP, Groen AK, Wagenaar E, van Deemter L (1993). Homozygous disruption of the murine mdr2 P-glycoprotein gene leads to a complete absence of phospholipid from bile and to liver disease. Cell.

[25] Al Inizi S, Gupta R, Gale A (2006). Fetal tachyarrhythmia with atrial flutter in obstetric cholestasis. Int J Gynaecol Obstet.

[26] Chan AL, Yu Lee H, Leung HW (2005). Fatal anaphylactic reaction to intravenous cephalexin. Clin Drug Investig.

[27] Gundling F, Tillmann HL, Schmidt O, Brennenstuhl M, Nerlich A, Schepp W (2005). [Severe intrahepatic cholestasis in a 66-year old male patient with medically treated atrial fibrillation]. Internist (Berl).

[28] O’Hare B (2008). A Case of Drug-induced Hepatotoxicity: Amiodarone is Not Always to Blame. The Med Forum.

[29] Li H, Durbin R (2009). Fast and accurate short read alignment with Burrows-Wheeler transform. Bioinformatics (Oxford, England).

[30] Li H, Handsaker B, Wysoker A, Fennell T, Ruan J, Homer N (2009). The Sequence Alignment/Map format and SAMtools. Bioinformatics (Oxford, England).

[31] DePristo MA, Banks E, Poplin R, Garimella KV, Maguire JR, Hartl C (2011). A framework for variation discovery and genotyping using next-generation DNA sequencing data. Nat Genet.

[32] Wang K, Li M, Hakonarson H (2010). ANNOVAR: functional annotation of genetic variants from high-throughput sequencing data. Nucleic Acids Res.

[33] Purcell S, Neale B, Todd-Brown K, Thomas L, Ferreira MA, Bender D (2007). PLINK: a tool set for whole-genome association and population-based linkage analyses. Am J Hum Genet.

